# Targeting cGAS-STING signaling: a potential therapeutic approach for the management of Huntington's disease

**DOI:** 10.17179/excli2024-8060

**Published:** 2025-01-24

**Authors:** Vishal Kumar, Puneet Kumar

**Affiliations:** 1 Department of Pharmacology, Central University of Punjab, Bathinda, India 151401

## ⁯⁯

Huntington's disease (HD) is a significant hereditary neurological condition in adults marked by several clinical manifestations, including depression, cognitive deterioration, and chorea (formerly referred to as Huntington chorea), commencing around the age of 40, with potential severity escalating by age 65. The estimated prevalence of this condition is 13-14 individuals per 100,000 persons globally. The primary areas of the brain affected in HD are the caudate nucleus and putamen, which constitute components of the striatum. Cortical pyramidal neurons and medium spiny neurons have heightened vulnerability to degeneration in HD due to their involvement in motor processes. The primary etiology of HD is a mutation in the Huntingtin gene (Htt), characterized by the increase of CAG (cytosine, adenine, guanine) triplet repeats in exon 1 of the Htt (Kumar et al., 2021[3]). As a result of the intricate nature of the disease, no long-term solution has been found; currently, the only options for treating HD symptoms include reversible vesicular monoamine transporter type-2 (VMAT-2) inhibitors Tetrabenazine and Deutetrabenazine, which have been approved by the US FDA (Claassen et al., 2022[1]). 

Neuroinflammation is the primary contributor to HD pathophysiology, driven by reactive gliosis, activation of astrocytes and microglia, and the creation of soluble inflammatory mediators. Microglia are the principal immune cells in the brain, constituting 5-10 % of the cells in the CNS. The principal role of microglia is the removal of microorganisms, deceased cells, protein aggregates, and other cellular detritus. Microglia monitor the CNS in a resting state and become activated upon detecting any pathogens or alterations in the microenvironment. The accumulation of mutant Huntingtin protein (mHTT) impairs microglial function, resulting in sustained activation. This microglial activation subsequently induces the production and release of cytokines (TNF-α, IL-6, and IL-10) and chemokines CCL2 (chemokine (C-C motif) ligand-2), CCL5 (C-C motif chemokine ligand 5), and CXCL10 (Chemokine (C-X-C motif) ligand 10), collectively fostering an inflammatory response that culminates in chronic inflammation and cell death. Chronic inflammation activates many pathways, including oxidative stress, mitochondrial malfunction, and excitotoxicity, leading to neuronal death. The literature extensively documents the expression of cytokines in the striatum and cerebral cortex of people with HD. Additionally, neuroinflammation compromises blood-brain barrier (BBB) permeability, facilitating the rapid ingress of immune cells and toxic chemicals into the brain, so exacerbating the neuroinflammatory response, and heightening neuronal susceptibility (Temgire et al., 2024[7]). 

DNA disruption, apoptosis, stimuli, mitochondrial damage, DNA viruses, and bacteria contribute to the generation of pathogen-associated molecular patterns (PAMPs) and danger-associated molecular patterns (DAMPs). PAMPs or DAMPs can be detected by pattern-recognition receptors (PRRs), which function as intrinsic cellular sensors that elicit a cellular stress response. Typically, hereditary DNA is confined to the nucleus and mitochondria, whereas cytosolic or extracellular DNA functions as PAMPs, activating DNA sensors to elicit innate immune responses. To counteract these harmful signals, cells possess various DNA sensors, including Toll-like receptor 9 (TLR-9), absent in melanoma 2 (AIM2), cyclic GMP-AMP synthase (cGAS), interferon gamma-inducible protein 16 (IFI16), DNA-dependent activators of IRFs, IFI165, and RNA polymerase III; however, only TLR-9, AIM2, and cGAS have been adequately characterized, while the others remain unverified (Hu et al., 2022[2]). 

The cGAS is an enzyme that synthesizes cyclic guanosine monophosphate-adenosine monophosphate (cGMP-AMP or cGAMP), a second messenger triggered by the binding of cGAS to DNA or RNA: DNA hybrids in the cytoplasm. cGAS can initiate signaling that promotes the upregulation of inflammatory genes and is pivotal in age-related macular degeneration and cellular senescence. cGAS-generated cGAMP interacts with the endoplasmic reticulum (ER)-associated transmembrane protein STING (stimulator of interferon genes), also referred to as TMEM173. STING recruits TANK-binding kinase 1 (TBK1), which phosphorylates transcription factors, including IFN regulatory factor 3 (IRF3) and IFN regulatory factor 7 (IRF7), as well as additional substrates such as IκB kinase α (IKKα), cRel, and p62 (sequestosome) (Verrier and Langevin, 2021[8]) (Supplementary information, Figure S1).

As a result, these substances penetrate the nucleus and initiate the production of type I interferons. Type-I interferons (IFNs) activate the Janus kinase (JAK)-signal transducer and activator of transcription (STAT) pathway to provoke an immunostimulatory response via the induction of interferon-stimulated genes (ISGs), leading to the release of proinflammatory cytokines and chemokines, such as TNF-α, IL-6, IL-1β, and the type-I IFNs themselves (IFN-α, IFN-β, and IFN-γ) (Paul et al., 2021[5]). 

A study by Ma et al. (2023[4]) found that the cGAS-STING pathway was active during neuroinflammation in Parkinson's disease-like MPTP mice. cGAS deficiency in microglia, rather than in peripheral immune cells, regulated neuroinflammation and neurotoxicity generated by MPTP. The mechanistic deletion of microglial cGAS mitigated neuronal dysfunction and the inflammatory response in astrocytes and microglia by suppressing antiviral inflammatory signaling. The injection of cGAS inhibitors provided neuroprotection to the animals following MPTP exposure. 

According to Sharma et al. (2020[6]), mouse HD striatal cells specifically raise cGAS mRNA, exhibiting elevated ribosome occupancy at exon 1, leading to enhanced cGAS protein production. cGAS is turned on by micronuclei in the cytoplasm of mouse HD striatal cells and human embryonic stem cell-derived HD neurons. The activation enhances inflammatory and autophagy responses via autophagy initiators LC3A and LC3B. Moreover, cGAS activity is elevated in the HD striatum, as shown by the heightened phosphorylation of STING and TBK1. These induce the upregulation of inflammatory genes, such as Ccl5 and Cxcl10. Conversely, cGAS reduction using CRISPR/Cas9 suppressed inflammatory gene expression and autophagy flux in HD striatal cells. 

In several neurodegenerative illnesses characterized by elevated neuroinflammation and heightened proinflammatory cytokine levels, suppression of the cGAS-STING pathway presents potential therapeutic opportunities. The STING inhibitor comprises nitrofuran derivatives (C-176 and C-178), nitro-fatty acids, naturally occurring chlorinated cyclopentapeptides (astin C and Aster tataricus derivatives), and tetradroisoquinolone acetic acids, which are reported to inhibit the palmitoylation of STING and diminish the binding affinity of cGAMP to STING. Conversely, cGAS inhibitors such as suramin, RU365, RU521, and PF-06928215 have been shown to impede its catalytic activity by obstructing cGAMP binding. Aminopyrimidines, Amlexanox, and GSK8612 inhibit the activation and release of IFN. They collectively decreased the generation of inflammatory cytokines and chemokines, thereby averting neuronal demise (Paul et al., 2021[5]). 

As a result, we hypothesize that inhibiting the cGAS-STING signaling pathway may serve as a novel therapeutic target for managing HD, as it promotes the microglial activation via the release of inflammatory cytokines, resulting in the production of more cytokines and chemokines, which in turn induces neuronal degeneration in the brain. We thoroughly reviewed the literature and analyzed the potential mechanisms driving cGAS-STING-driven HD pathogenesis. As a result, developing powerful cGAS-STING inhibitors that are selective and multitargeted may provide an innovative treatment approach for HD. We recommended that more studies be conducted to clarify the precise mechanism of cGAS-STING in HD.

## Conflict of interest

The authors declare there is no conflict of interest.

## Supplementary Material

Supplementary information
