# Supplementary material for: Targeting cGAS-STING signaling: a potential therapeutic approach for the management of Huntington's disease
Source: EXCLI J. 2025 Jan 24;24:201–3. doi: 10.17179/excli2024-8060 (PMC11897795; doi:10.17179/excli2024-8060)
Supplement: Supplementary information [file EXCLI-24-201-s-001.pdf]

EXCLI Journal 2025;24: 201-203 – ISSN 1611-2156
Received: November 28, 2024, accepted: January 20, 2025, published: January 24, 2025

Supplementary information to:

Letter to the editor:

TARGETING cGAS-STING SIGNALING: A POTENTIAL THERAPEUTIC APPROACH FOR THE MANAGEMENT OF HUNTINGTON'S DISEASE

Vishal Kumar, Puneet Kumar*

Department of Pharmacology, Central University of Punjab, Bathinda, India 151401

* **Corresponding author:** Dr. Puneet Kumar, Professor, Department of Pharmacology, Central University of Punjab, Bathinda, India 151401. E-mail: punnubansal79@gmail.com

https://dx.doi.org/10.17179/excli2024-8060

This is an Open Access article distributed under the terms of the Creative Commons Attribution License (http://creativecommons.org/licenses/by/4.0/).

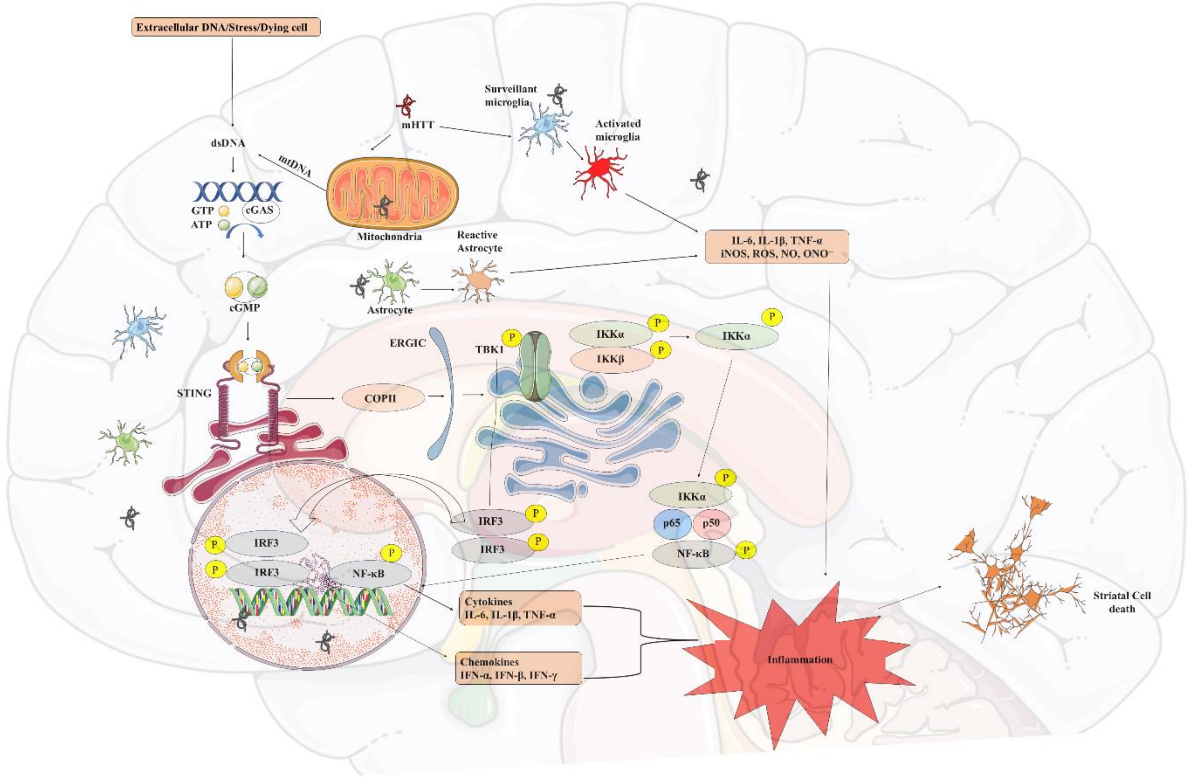

Supplementary Figure 1: Schematic representation of the cGAS-STING pathway in the pathogenesis of Huntington's disease. cGAS interacts with mitochondrial DNA released by mHTT, extracellular and DNA released from apoptotic cells, to synthesize cGAMP (a secondary messenger) from ATP and GTP. cGAMP also connects to and turns on STING dimers on the endoplasmic reticulum.

EXCLI Journal 2025;24: 201-203 – ISSN 1611-2156
Received: November 28, 2024, accepted: January 20, 2025, published: January 24, 2025

These dimers then join with coatomer protein complex II (COPII) vesicles and move through the ER-Golgi intermediate compartment (ERGIC) to the Golgi apparatus. In the Golgi apparatus, STING recruits TANK-binding kinase-1 (TBK1), facilitating TBK1 autophosphorylation and the recruitment of interferon regulatory factor-3 (IRF3). The phosphorylation of IRF3 by TBK1 facilitates IRF3 dimerization and its translocation to the nucleus, hence promoting the production of type 1 interferon, along with several inflammatory mediators and chemokines. TBK1 activates the inhibitor of nuclear factor β kinase (IKK), which phosphorylates IκBα, therefore alleviating the inhibition of nuclear factor kappa B (NF-κB), consisting of p65 and p50 subunits. NF-κB translocates to the nucleus, where aggregated mHTT promotes NF-κB, resulting in the induction of inflammatory cytokines production. In addition to this, cytoplasmic mHTT stimulates astrocytes and microglia, leading to the release of cytokines and the formation of free radicals, including reactive oxygen species (ROS). Collectively these factors contribute in neuroinflammation which leads to striatal cell death.
